# Exact and Ubiquitous Condition for Solid-State Deracemization in Vitro and in Nature

**DOI:** 10.1021/jacs.3c11332

**Published:** 2024-02-02

**Authors:** Leif-Thore Deck, Mercedeh Sadat Hosseinalipour, Marco Mazzotti

**Affiliations:** Institute of Energy and Process Engineering, ETH Zurich, Zurich 8092, Switzerland

## Abstract

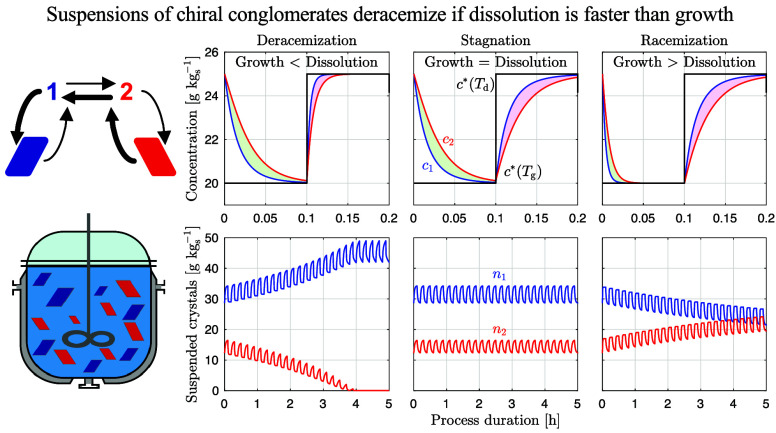

Solid-state deracemization is the amplification of an enantiomeric excess in suspensions of conglomerate-forming chiral compounds. Although numerous chemical and biochemical compounds deracemize, its governing mechanism has remained elusive. We introduce a novel formulation of the classical population-based model of deracemization through temperature cycles to prove that suspensions deracemize whenever a simple and ubiquitous condition is met: crystal dissolution must be faster than crystal growth. Such asymmetry is a known principle of crystallization, hence explaining the generality of deracemization. Through both experiments and a theoretical analysis, we demonstrate that this condition applies even for very small temperature cycles and for random temperature fluctuations. These findings establish solid-state deracemization as an attractive route to the manufacture of enantiopure products and as a plausible pathway toward the emergence of homochirality in nature.

## 1 Introduction

Solid-state deracemization refers to the amplification of an enantiomeric excess in suspensions of conglomerate-forming compounds in the presence of racemization in solution.^1−4^ It is an attractive route to the manufacture of enantiopure products and a possible pathway to the emergence of homochirality on Earth,^2,4,5^ which is linked to the origin of life.^6,7^ It has been demonstrated by various methods of manipulating the crystalline suspension, namely, isothermal grinding or milling,^2,4^ the application of ultrasound,^8,9^ temperature-cycling in a single^10,11^ or in two coupled vessels,^12,13^ high-pressure homogenization,^14^ and solvent-cycling.^15,16^ Numerous compounds have been shown to deracemize, including many of biological relevance,^17−19^ which suggests the existence of a general governing mechanism.^2−4,8−11,14−16,20,21^ Yet, pinning down such a mechanism has been difficult, controversial, and not conclusive.^22−25^ In this work, we introduce a simplified formulation of the classical population balance model of deracemization through temperature cycles to prove an exact condition under which deracemization occurs: crystal dissolution must be faster than crystal growth. Such kinetic asymmetry is a fundamental principle of crystallization,^26−28^ which explains the general success of the deracemization experiments reported in the literature.

The analysis that follows comprises theory, numerical simulations, and experiments. In Section 2.1, the mechanism of solid-state deracemization by temperature cycling is conceptualized, which serves as a starting point for the derivation of an exact condition for deracemization reported in Section 2.2. Comprehensive numerical simulations presented in Section 2.3 confirm that this condition applies not only to deracemization with periodic temperature cycling but also to processes with arbitrarily oscillating temperature profiles. These findings are validated experimentally: Section 2.4 reports the results of deracemization experiments with the chiral compound *N*-(2-methylbenzylidene)-phenylglycine amide (NMPA) carried out using periodic temperature cycles, uncontrolled ambient thermal conditions, and a tightly controlled constant temperature level. Finally, conclusions are drawn, and implications for the emergence of homochirality in nature are discussed in Section 3.

## 2 Results and Discussion

### 2.1 Conceptual Analysis

We model solid-state deracemization using population balance equations (PBEs), an approach widely applied in industrial crystallization and related fields.^29−31^ Through numerical simulations, our group has successfully described deracemization for both temperature-cycling and isothermal process variations (the latter commonly termed Viedma ripening).^24,32−34^ These earlier models attribute deracemization to the interplay between enantioselective crystal agglomeration (yielding a larger crystal by the merger of two smaller ones),^31^ crystal breakage and attrition, and crystal ripening (the preferential dissolution of smaller crystals due to size-dependent solubility).^30,35,36^ Even though the overall mechanism of deracemization remains elusive, most contributions agree that both crystal agglomeration and ripening play pivotal roles in it.^22−25^

The mechanistic character of population balance-based models implies that they comprise numerous parameters, some of which are neither accessible through experiments nor predictable through theory. The identification of accurate rate expressions for crystal agglomeration, breakage, and ripening, as well as the ensuing estimation of the kinetic parameters from experimental data, has been found to be particularly challenging. For this reason, it has not yet been possible to conclusively demonstrate which mechanisms control solid-state deracemization. Here, we tackle this challenge by reducing the complexity of the underlying population balance model: we eliminate all elements that we prove are not essential to deracemization, thus enabling the derivation of an analytical solution and the design of ad hoc experiments to confirm the theoretical findings.

In the general model, we consider deracemization through periodic temperature cycling in a well-stirred batch crystallizer (*i* = 1, 2 and *j* = 3 – *i*), where the target (major) enantiomer is *i* = 1, and the undesired (minor) enantiomer is *i* = 2. The racemization reaction between the two enantiomers (E_1_ and E_2_) in solution is described as a reversible first-order chemical reaction
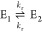
1where the temperature-dependent reaction rate constant, *k*_r_(*T*), is the same in both directions because of symmetry; therefore, at chemical equilibrium, the concentration of the two enantiomers is obviously the same. The material balance is
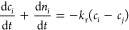
2where *c*_*i*_ and *n*_*i*_ denote the mass of the solute per unit mass solvent in solution and in the solid phase, respectively. The quantity *n*_*i*_ is given in terms of the particle size distribution of the *i*th enantiomer crystals, *f*_*i*_ (PSD), as
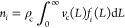
3where ρ_c_ is the crystal density and *v*_c_(*L*) = *k*_v_*L*^3^ is the volume of a crystal of characteristic size *L* and volume shape factor *k*_v_. It is worth noting that, in the model system, enantiomers are present either as molecular species in solution (i.e., monomers) or as enantiopure crystals but not as chiral clusters (i.e., oligomers), as proposed in other studies.^22,25^

Equation 2 is coupled to the PBEs that determine the two PSDs *f*_*i*_(*t*, *L*); these are transient integro-differential equations that account in general for crystal agglomeration and breakage (both through integral terms) and for crystal growth and dissolution (through a differential term). In the context of deracemization, nucleation is neglected for two reasons. First, the classical inclusion of nucleation through the boundary condition of the PBE at *L* = 0 is incompatible with a model accounting for size-dependent solubility, unless master equations are used that lead to an excessive computational burden.^37^ Second, experiments are typically carried out under conditions where little to none nucleation takes place; in particular, the use of low cooling rates and of high solid loadings prevents the suspension from attaining the high supersaturation levels that would trigger nucleation.^10,11^

The temperature in the crystallizer is assumed to be precisely controlled, undergoing periodic cycles, each consisting of a cooling ramp from high temperature, *T*_d_, to low temperature, *T*_g_, a holding period, *t*_g_, at *T*_g_ (termed growth step), a heating ramp, and a holding period, *t*_d_, at *T*_d_ (termed dissolution step). In the following, heating and cooling ramps are replaced by step changes between temperature levels, without loss of generality. Such a cycle is associated with a solubility difference Δ*c*_∞_ = c*(*T*_d_) – c*(*T*_g_), where c*(*T*) is the solubility of a crystal of infinite size, i.e., the value that is used when the size-dependency of solubility is neglected.

**Figure 1:**
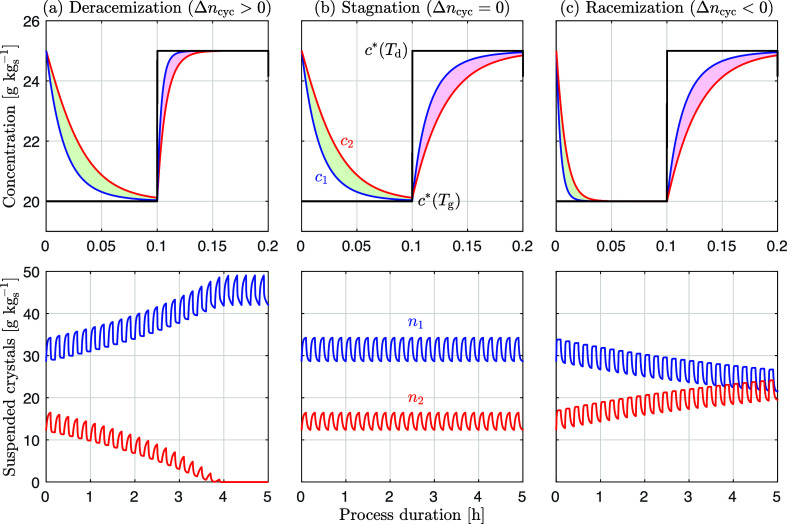
Simulations of temperature cycling-induced deracemization for three cases with different process behaviors. Top row: evolution of concentration levels in solution during a single cycle. Bottom row: evolution of suspended crystal density during the entire process. The three simulations were generated by using identical model parameters except for the relative rates of growth and dissolution. Growth is either slower than (a), as fast as (b), or faster than (c) dissolution.

To assess whether deracemization occurs or not, different indicators can be used, either the change of crystal mass of the two enantiomers over one cycle or the extent of conversion reaction from the minor to the major enantiomer during one cycle. The change in mass of the major enantiomer crystals per unit mass of solvent throughout a single cycle, Δ*n*_cyc_, is defined as
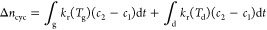
4whereby the boundaries of the integrals (g,d) refer to the growth and dissolution steps, respectively. Note that Δ*n*_cyc_ > 0 is required for successful deracemization. This is illustrated in Figure 1, whose three panels illustrate process simulations of temperature cycling (see Section 4.1.2) for different relative rates of dissolution and growth, with all other model parameters being identical. In order to highlight the mechanisms that are prerequisite for deracemization, neither crystal agglomeration, nor breakage or attrition, nor ripening have been included. The upper row shows the evolution of the concentration levels *c*_1_ (blue, major enantiomer) and *c*_2_ (red, minor enantiomer), as well as the value of the solubility c* (black) during a single temperature cycle. The lower row shows the evolution of the crystal mass suspended, *n*_*i*_, for both crystal populations during multiple cycles.

Crystals grow at low temperature and dissolve at high temperature. During both steps, a difference in concentration between the enantiomers emerges (shaded areas), which drives the racemization reaction (see eq 2) and governs the process performance. Deracemization happens when the net effect of the racemization reaction throughout a temperature cycle favors the major enantiomer (i.e., when Δ*n*_cyc_ > 0, see eq 4). To enable a visual analysis of deracemization, we neglect the temperature dependence of *k*_r_(*T*) in Figure 1, so that the shaded areas are proportional to the amount of reacted material (the integrals in eq 4). In (a), where model parameters are such that dissolution is fast and growth is slow (in general terms, that will be made more precise in the next section), the green area (proportional to the extent of conversion of the minor enantiomer into the major enantiomer) is larger than the red area (proportional to the extent of conversion of the major enantiomer into the minor enantiomer); hence, the net effect during the whole cycle favors the major enantiomer and deracemization occurs (Δ*n*_cyc_ > 0). In panel (c), on the contrary, where growth is fast and dissolution is slow, the red area is larger than the green one; hence, temperature-cycling racemizes the suspension (Δ*n*_cyc_ < 0). In panel (b), where dissolution and growth have similar rates, both areas are of equal size, and temperature cycling does not alter the handedness of the suspension (Δ*n*_cyc_ = 0).

It follows that deracemization is successful if and only if dissolution is faster than growth (in general terms), even in the absence of crystal agglomeration, crystal breakage or attrition, and crystal ripening. This is a novel and simple criterion for deracemization, which is demonstrated rigorously in the next section and whose consequences are presented and demonstrated in the sections after the next.

### 2.2 Exact Condition for Deracemization

The model used in the previous section, i.e., with neither breakage nor agglomeration nor size-dependent solubility, has been further simplified for the analysis that follows. First, the area of the active surface of the crystals, i.e., the area where growth and dissolution take place, is assumed to remain constant as the volume of the crystals changes. This would be the case for rods that grow and dissolve only in the length direction; it would also apply to arbitrary crystal geometries if the actual surface area change through a temperature cycle is small. Second, the growth and dissolution rate of crystals of type *i* is given by a temperature-dependent rate constant, *k*_m_(*T*_m_) (the subscript m = g or m = d indicates growth or dissolution, respectively), multiplied by the linear driving force *x*_*i*_ = *c*_*i*_ – c*(*T*_m_). Thorough numerical simulations have proven that these two assumptions can be relaxed without changing the main conclusions below (see Supporting Information, Section S.2).

Under these assumptions, the model reduces to two linear ODEs that can be written in vector notation (see methods Section 4.2 for the detailed derivation) as
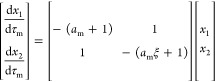
5where the matrix of coefficients of the linear system is called , and τ_m_ = *tk*_r_(*T*_m_) is a dimensionless time. The dimensionless parameter *a*_m_ = 3ρ_c_*k*_v_*m*_2,1_*k*_m_(*T*_m_)/*k*_r_(*T*_m_) is proportional to the ratio of the rate constant of growth or dissolution and of the racemization reaction (at the relevant temperature); *m*_2,*i*_ denotes the second moment of the *i*th PSD, which scales with the population’s surface area per unit mass of solvent; the quantity ξ = *m*_2,2_/*m*_2,1_ < 1 characterizes the asymmetry between the enantiomers (the simplest case is where the two crystal populations are similar in size and shape, but the amount of the minor enantiomer crystals is smaller than that of the major enantiomer crystals). Implementing temperature cycles implies solving the system above for 0 ≤ τ ≤ τ_g_ = *k*_r_(*T*_g_)*t*_g_ for an initial solution composition *x*_*i*_^0^, typically racemic, so as *x*_*i*_ becomes *x*_*i*_^g^; then switching to the dissolution temperature level and setting *x*_*i*_^0^ = *x*_*i*_^g^ – Δ*c*_∞_ (so as to account for the change in solubility) and solving the new version of the system above for 0 ≤ τ ≤ τ_d_ = *k*_r_(*T*_d_)*t*_d_; and finally switching back to *T*_g_ and setting *x*_*i*_^0^ = *x*_*i*_^d^ + Δ*c*_∞_. In this way, the evolution of the solution composition is obtained, depending on the six model parameters *a*_g_, *a*_d_, Δ*c*_∞_, ξ, τ_g_, and τ_d_.

Independent of the initial state, the solution composition reaches a cyclic steady state, as long as the surface areas of crystals and thus the parameter ξ do not change (see Supporting Information, Section S.2.2). The net amount of the minor enantiomer converted into the major enantiomer in eq 4, Δ*n*_cyc_, can be calculated in a closed form as
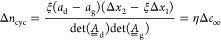
6where the matrices  and  are implicitly defined through eq 5, and Δ*x*_*i*_ is the change in the concentration of enantiomer *i* during the dissolution step given in a closed form by
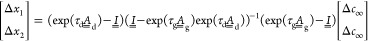
7where  is the matrix exponential and  is the unitary matrix. The second equality in eq 6 introduces the cycle efficiency, η, which is the ratio between Δ*n*_cyc_ and the solubility difference Δ*c*_∞_, i.e., the maximum value of Δ*n*_cyc_ that a single cycle enables; η is independent of the amplitude of the temperature cycles because Δ*x*_*i*_ scales with Δ*c*_∞_ as to eq 7. The two determinants in eq 6 are always positive, as also ξ obviously is (ξ = 0 corresponds to an enantiopure suspension). We found that also the term (Δ*x*_2_ – ξΔ*x*_1_) in the numerator of eq 6 is always positive by evaluating it for a huge number of combinations of model parameters, selected randomly within broad ranges of values.

Therefore, we conclude that the cycle efficiency η is positive and deracemization occurs if and only if
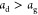
8or, as to the relevant definitions, if and only if
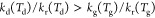
9

Remarkably, this condition is independent of the duration of the dissolution and growth phases, τ_d_ and τ_g_, of the enantiomeric asymmetry, ξ, and of the solubility difference, Δ*c*_∞_. This implies that the amplitude of the temperature oscillations leading to successful deracemization may even be extremely small (see Section 2.4 where the reported successful deracemization experiments at a controlled temperature exhibit a standard deviation of 0.02 °C) and that the result above applies not only to deracemization by temperature cycling but also to isothermal Viedma ripening, where minimal temperature fluctuations may certainly be caused by intense stirring or by grinding. The analysis applies also when temperature cycling is replaced by periodic removal and readdition of the solvent at constant temperature, as proposed in recent experimental studies.^15,16^

Note that in practice and under rather general conditions, crystal dissolution is faster than growth for the same thermodynamic driving force and at the same temperature.^26−28^ This is because the crystal shape evolves during growth toward a steady-state shape dominated by the slowest-growing crystal facets.^26,38,39^ Yet, the opposite occurs during dissolution, when the crystal shape evolves away from the steady-state shape, thus exposing fast-dissolving facets. Data reported in the literature indicate that for instance *k*_d_/*k*_g_ = 4 for sodium chlorate,^28^ and *k*_d_/*k*_g_ > 2.5 for paracetamol^27^ (consider Snyder and Doherty^26^ for a detailed discussion of the asymmetry between growth and dissolution).

Although a strong temperature dependence of the racemization rate constant may in theory switch the sign of the inequality in eq 9, in practice, this is very unlikely given the relatively small amplitudes of temperature cycles used in experiments (order of 5–20 °C),^10,11,40^ if not at all impossible when considering the very small-amplitude temperature-cycling experiments presented in this work (see Section 2.4).

Finally it is worth noting that the condition derived above holds true no matter how slow (or fast) the chemical reaction is (see Figure S1 in the Supporting Information). This is an important remark because most chiral compounds do not racemize easily. For example, amino acids in solution racemize very slowly at ambient temperature and neutral pH, i.e., with a characteristic time on the order of thousands of years.^41,42^ The amount of material that reacts in such a case during a temperature-cycle with characteristic times for growth and dissolution on the order of minutes to hours is very small, and deracemization consequently proceeds only very slowly. From an industrial manufacturing perspective, the implementation of solid-state deracemization is particularly interesting for compounds with fast racemization, e.g., for NMPA that racemizes in the presence of non-nucleophilic bases within time scales of 10 min to an hour^4,43^ (see Section 2.4).

Based on the considerations above, we argue that the condition in eqs 8 and 9 is met for most chiral species that crystallize as conglomerates and that racemize (perhaps even for all), in a broad range of temperatures. Thus, solid-state deracemization is based on a simple and ubiquitous growth-dissolution mechanism that requires neither grinding nor agglomeration nor ripening.

### 2.3 Extension to Nearly Isothermal Conditions

While the exact condition above was derived for deracemization through temperature cycles that induce a periodic change of solubility, here, we assess through simulations carried out using the PBE model presented in Section 4.1 to what extent such a condition and the underlying growth-dissolution mechanism apply also to isothermal deracemization, i.e., to Viedma ripening, as conjectured in the previous section.

**Figure 2:**
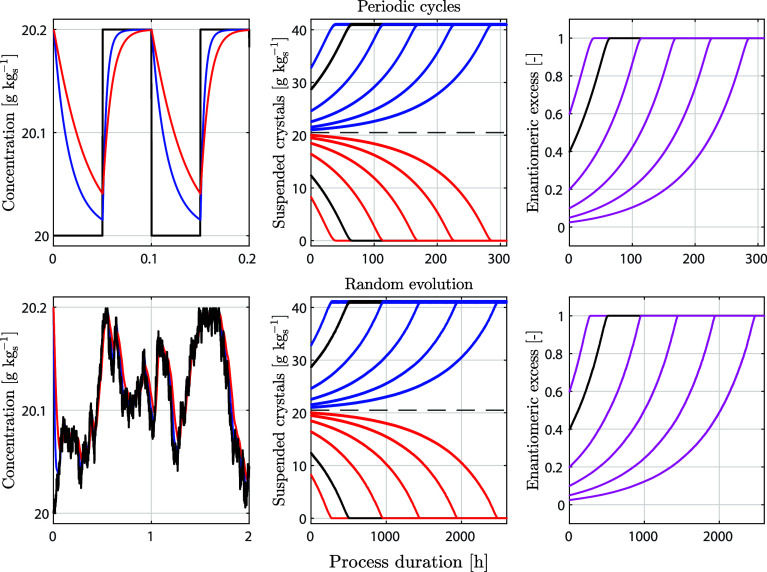
Simulations of nearly isothermal deracemization processes. The top row shows the case of small periodic temperature cycles, and the bottom row the case of random temperature fluctuations, generated as random walk constrained between the solubilities used in the periodic cycles. The left panels show the concentration in solution for the simulation with ξ = 0.43, which is the value also used in the experiments. The center panels show the corresponding evolution of the crystalline mass and the right ones that of the enantiomeric excess with different initial values of ξ (black lines correspond to ξ = 0.43).

Figure 2 shows the outcome of simulations of two processes with *a*_d_/*a*_g_ = 4 and different initial values of ξ. In the first case (top), the suspension undergoes temperature cycles where the solubility changes by 1%, corresponding to a temperature differential of about 0.2 °C for the compound used in the experiments reported below. In the second case (bottom), the suspension is subject to random temperature fluctuations generated through a random walk constrained within the same range of solubilities; the mean rate of temperature change equals that of the periodic cycling. Similar to Figure 1, the left panels illustrate the evolution of the concentration levels in solution and the center ones those of the crystals in suspension; in addition, the right panels indicate the evolution of the enantiomeric excess in the suspension, which is defined as ee = (*n*_1_ – *n*_2_)/(*n*_1_ + *n*_2_) and quantifies enantiopurity.

Notably, deracemization is achieved in both types of simulations, whereby it is faster for periodic temperature cycles than for random fluctuations. In all simulations, the temporal evolution of the enantiomeric excess exhibits an acceleration over time. Such autocatalytic behavior has been observed in several experimental studies on Viedma ripening in the literature;^4,40,44^ it has been argued that nonlinear phenomena such as crystal agglomeration are required to explain both deracemization and the mentioned acceleration.^2,3,22−25^

Here, we offer a completely different explanation. First, we crucially conjecture that any supposedly isothermal experiment still exhibits arbitrary temperature fluctuations, particularly when a suspension is subject to intense stirring or grinding, as in Viedma ripening experiments; therefore, the random temperature fluctuation simulations presented here are indeed representative of Viedma ripening conditions. Second, such simulations demonstrate that deracemization, both in the temperature-cycling case and in the Viedma ripening case, is achieved without including any nonlinear phenomenon, while the acceleration in the evolution toward deracemization is due to the fact that the enantiomeric ratio ξ decreases over time because of the conversion of the minor enantiomer into the major one; this leads to an increase over time of the cycle efficiency, η, until the deracemization rate becomes controlled by the irreversible disappearance of crystals of the minority enantiomer during the dissolution steps (see Supporting Information, Section S.1.3).

### 2.4 Experimental Evidence

**Figure 3:**
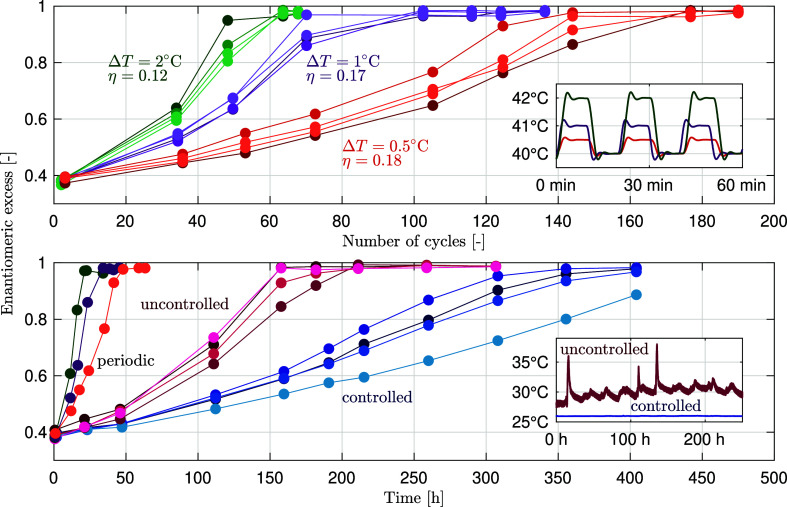
Evolution of the enantiomeric excess for deracemization experiments using NMPA with an initial enantiomeric excess of 0.4 (ξ = 0.43); all repetitions of each experiment are shown. Top panel: temperature-cycling experiments with small amplitude, i.e., of 2 °C (green), 1 °C (violet), and 0.5 °C (orange) plotted in terms of the number of cycles. Bottom panel: controlled (constant temperature, blue) and uncontrolled (random temperature fluctuations, red) experiments plotted in terms of time; one representative experiment for each of the periodic temperature cycling experiment is shown for comparison. In both panels, the insets show the thermal evolution for the relevant experiments, as indicated.

To provide experimental evidence of the theoretical results above, we carried out deracemization experiments with the conglomerate-forming species NMPA, in the presence of the base 1,8-diazabicyclo[5.4.0]undec-7-en (DBU) that catalyzes its racemization in solution, using 10 mL glass vials (see details in Section 4.3). The evolution of the enantiomeric excess during these experiments is shown in Figure 3.

The first set of experiments (top panel) consists of temperature cycling with very small amplitudes, i.e., of 2, 1, and 0.5 °C, using a magnetic bar for stirring and with an initial asymmetry of ξ = 0.43 (corresponding to ee = 0.40); complete deracemization is achieved in ca. 55, 80, and 160 cycles, respectively (i.e., between 0.5 and 2 days). This is consistent with the model-based analysis above. The number of cycles must in fact scale with the reciprocal of Δ*n*_cyc_ in eq 4; therefore, with the reciprocal of the solubility difference, Δ*c*_∞_ (proportional to the temperature difference for small amplitudes), and with the reciprocal of the cycle efficiency, η (largely independent of the temperature difference). To confirm this, we computed the value of η following the approach outlined in Supporting Information, Section S.1 not only for these three experiments where we found 0.12 ≤ η ≤ 0.18 but also for a large set of earlier NMPA temperature-cycling experiments with similar operating conditions and an amplitude up to 21 °C where we found 0.08 ≤ η ≤ 0.13^45^ (see Supporting Information, Section S.1 for the details, as well as for consistent results obtained for two other chiral compounds).

Then, we carried out two additional sets of deracemization experiments in the same equipment, whose outcome is shown in the bottom panel of Figure 3. The first was operated at 26 °C in a tightly controlled manner (the average temperature was 25.97 °C, with a standard deviation of 0.02 °C). The second was operated under uncontrolled conditions (i.e., with no temperature control), thus allowing for temperature fluctuations caused by the varying room conditions in the lab. The uncontrolled experiments deracemized faster than the controlled ones (about 8 days instead of more than 15 days), which is justified by the presence of larger temperature fluctuations mostly between 28 and 30 °C; in fact, the 24 h period of day–night temperature fluctuations can be easily recognized in the associated temperature profile.

A few remarks are worth making. First, the experiments with small-amplitude periodic oscillations confirm the conclusions derived from the theoretical approach above. Second, the controlled experiments confirm that Viedma ripening, i.e., deracemization attained at constant temperature typically by continuously grinding the suspension, is explained by the same growth/dissolution mechanism of deracemization via temperature cycles, when the unavoidable random temperature fluctuations are accounted for.

Third, the uncontrolled experiments, with random temperature fluctuations, as illustrated in the bottom panel of Figure 3, have obvious implications on theories about the emergence of homochirality in nature: since tiny, random temperature fluctuations enable deracemization, any kind of arbitrary temperature profiles experienced by prebiotic systems (because of daily and seasonal temperature variability) may have led to deracemization and to homochirality.

## 3 Conclusions

This work introduces and validates a general mechanism for solid-state deracemization based on growth and dissolution driven by temperature cycling, either periodically or randomly, even with very small temperature amplitudes. Through the derivation of an analytical solution, we obtained an exact condition for deracemization: suspensions of conglomerate crystals in the presence of a racemization reaction in solution deracemize when crystal dissolution is faster than crystal growth in the terms discussed above. Such a condition is ubiquitous;^26−28^ hence, solid-state deracemization of conglomerate-forming chiral compounds provides both a convenient route toward enantiopure products, e.g., in the pharmaceutical sector where this is a major challenge, and a natural pathway to amplify asymmetries in enantiomeric composition all the way to homochirality.

Numerous biorelevant compounds such as the amino acids threonine and asparagine crystallize as conglomerates^17^ and hence are candidates for solid-state deracemization. Amino acids in solution racemize even at ambient temperature and neutral pH, albeit slowly (order of thousands of years), and they do so many orders of magnitude faster at high temperatures and low pH.^41,42^ Since even small, random temperature fluctuations (or the natural day–night cycle) enable deracemization, homochiral suspensions of these compounds may well have emerged in prebiotic environments. This is in stark contrast to the main competing theoretical mechanism for chiral amplification, i.e., asymmetric autocatalysis, for which only a single example (in fact with no prebiotic relevance) has ever been found experimentally.^46,47^ The initial asymmetry required to kick-off deracemization may have been generated in multiple ways,^48^ yet a certain asymmetry is intrinsic to crystallization due to the inherent stochasticity of the underlying microphysical phenomena such as nucleation.^1,49,50^ Based on the simple and ubiquitous mechanism demonstrated in this work, we argue that solid-state deracemization may indeed have played a pivotal role in the origin of homochirality on Earth.

## 4 Materials and Methods

Here, we present the general PBE model (Section 4.1), the simplified model, the analytical derivation of the exact condition for deracemization (Section 4.2), and the experimental methods (Section 4.3).

### 4.1 General PBE Model

#### 4.1.1 Model Equations

We model deracemization in a well-stirred batch crystallizer (*i* = 1, 2 and *j* = 3 – *i*), where the target (major) enantiomer is *i* = 1, and the undesired (minor) enantiomer is *i* = 2. The material balance is
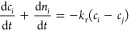
10with *c*_*i*_ and *n*_*i*_ the mass of the solute per unit mass solvent in solution and in the solid phase, whereby
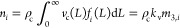
11and *k*_r_ is the temperature-dependent rate constant of racemization and *v*_c_(*L*) = *k*_v_*L*^3^ is the volume of an individual crystal of size *L* and volume shape factor *k*_v_. Equation 10 is coupled to the PBEs of the two populations. The *k*th moment of the particle size distributions of enantiomer *i* is defined as
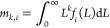
12

#### 4.1.2 Numerical Simulations

We numerically implemented the generalized model presented in Section 4.1.1 using Matlab R2022b. All simulations were carried out for monodisperse populations with crystals of the size *L*_*i*_(*t*). This is for two reasons; first, the effect of polydispersity on deracemization has been studied extensively in an earlier contribution to which we refer the interested reader.^34^ Second, the newly introduced deracemization mechanism is largely independent of the shape of the particle size distribution; hence, little insight can be obtained from the analysis of more complex polydisperse systems. The *k*th moment of the PSDs of enantiomer *i* thus is
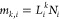
13where *N*_*i*_ is the number of crystals of enantiomer *i* per mass of solvent. Since here we consider neither agglomeration, breakage, nor nucleation, this number is constant throughout the entire process, until homochirality is reached and the population of enantiomer 2 dissolves, at which point *N*_2_ = 0. The initial state of the suspension is characterized through initial sizes *L*_0,*i*_ and numbers *N*_0,*i*_ of the two enantiomers. All simulations employ *N*_0,2_ < *N*_0,1_ as a source of the initial asymmetry and *L*_0_ = *L*_0,1_ = *L*_0,2_, and they start with a growth step. We consider an equal initial size for two reasons: first, in actual experiments, the initial crystal sizes of both enantiomers are rather similar. Second, a difference in the mean crystal size is primarily relevant in the context of crystal ripening (which is faster for smaller crystals), but this is a phenomenon not essential to the new mechanism. Thanks to the monodispersity, the model reduces to a set of four ODEs that describe the evolution of *c*_1_, *c*_2_, *L*_1_, and *L*_2_
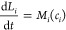
14
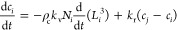
15where *M*_*i*_ equals *G*_*i*_ during growth steps and *D*_*i*_ during dissolution steps and  (termed 3D growth). For simulations carried out under the assumption also used in the derivation of the analytical solution that the second moment of the PSD remains constant during temperature cycling, we impose  (termed 1D growth). In this case, the system simplifies into a set of two ODEs (Section 4.2). In terms of the kinetic rate expressions for crystallization, we consider the following two cases
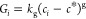
16
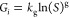
17whereby the dissolution rates are defined analogously. Note that the dimension of the rate constant depends on both the choice of the driving force and the value of the exponent. The saturation ratio *S*_*i*_ is defined as (neglecting activity coefficients)
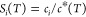
18

From a thermodynamic point of view, the logarithmic expression of the driving force is the most accurate. Yet, given that the typical operating conditions of deracemization experiments do not involve particularly high super- or undersaturation levels, we consider it adequate to linearize the logarithm when deriving the analytical solution, which yields a driving force based on the concentration difference. We confirmed the applicability of this assumption by comparing numerical simulations carried out using both linear and logarithmic driving forces, as presented in Supporting Information, Section S.2.

A complete list of the simulation parameters is provided in Table 1. In general, parameter values were chosen to match the behavior of the model compound NMPA, which has been studied extensively in earlier contributions on deracemization through both temperature cycling^11,43,45^ and high-pressure homogenization.^14^

**Table 1:** List of Simulation Parameters, Grouped into Physicochemical Quantities, Process Conditions, and Numerical Parameters^a^

parameter	symbol	values [unit]
crystal density	ρ_c_	1300 [kg m^–^^3^]
solvent density	ρ_s_	786 [kg m^–^^3^]
volume shape factor	*k*_v_	π/4 [–]
growth rate exponent	*g*	1 [–]
dissolution rate exponent	*d*	1 [–]
growth prefactor at *T*_g_ (Δ*c* driving force)	*k*_g_(*T*_g_)	10^–4.699^ [m s^–1^ kg^–1^ kg_s_]
growth prefactor at *T*_g_ (ln (*S*) driving force)	*k*_g_(*T*_g_)	10^–6.398^ [m s^–1^]
growth/dissolution ratio	*k*_d_(*T*_g_)/*k*_g_(*T*_g_)	4 [–]
reaction rate constant at *T*_g_	*k*_r_(*T*_g_)	0.2 [min^–1^]
temperature-dependency of dissolution	*k*_d_(*T*_d_)/*k*_d_(*T*_g_)	1 [–]
temperature-dependency of reaction	*k*_r_(*T*_d_)/*k*_r_(*T*_g_)	1 [–]
		
solubility at *T*_g_	c*(*T*_g_)	20 [g kg_s_^–1^]
solubility at *T*_d_	c*(*T*_d_)	20.2 or 25 [g kg_s_^–1^]
number density of crystals	*N*_0_ = *N*_0,1_ + *N*_0,2_	10^8^ [kg_s_^–1^]
initial crystal size	*L*_0_ = *L*_0,1_ = *L*_0,2_	10^–4^ [m]
initial enantiomeric ratio	ξ	0.43 [–]
time of growth step	*t*_g_	variable
time of dissolution step	*t*_d_	variable
initial concentration	*c*_0_ = *c*_0,1_ = *c*_0,2_	c*(*T*_d_)
		
simulation time step	*t*_step_	1 [s]
process duration	*t*_tot_	variable, until *N*_2_ = 0

a The reported parameter values represent the base case. If specific simulations use different values, this is indicated elsewhere. Note that an initial enantiomeric ratio of ξ = 0.43 corresponds to an initial enantiomeric excess of ee = 0.40, which was also used in the experiments.

For the generation of Figure 1, cases were simulated in which growth and dissolution occur at similar rates (panels b) and where growth is faster than dissolution (panels c). In case (b), it holds that *k*_g_(*T*_g_) = *k*_d_(*T*_d_) = 10^–4.699^ m s^–1^ kg^–1^ kg_s_, whereas in case (c), it holds that *k*_g_(*T*_g_) = 4 × 10^–4.699^ m s^–1^ kg^–1^ kg_s_ and *k*_d_(*T*_d_) = 10^–4.699^ m s^–1^ kg^–1^ kg_s_.

### 4.2 Simplified Model

#### 4.2.1 Simplified Model Equations

The expressions for *n*_*i*_ and its derivative simplify when assuming no nucleation and a linear driving force for growth and dissolution as follows
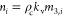
19
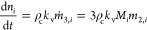
20where *M*_*i*_ is the rate of growth or dissolution (depending on the step) of enantiomer *i*, for which we assume a linear driving force
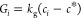
21
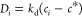
22

We discuss the effect of a nonlinear driving force in Supporting Information, Section S.2. c* is the solubility at the relevant temperature, and *k*_m_ is the rate constant of either growth or dissolution. Note that both solubility and crystallization kinetics are identical for both enantiomers and that the solubility of each enantiomer is assumed to be independent of the concentration of the second enantiomer, i.e., the solution is ideal.

We next introduce the assumption that the second moments of the PSDs do not change during temperature cycling, i.e., the crystals’ active surface area remains constant as their volume changes, so that the model reduces to two linear ordinary differential equations
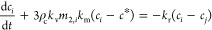
23

Such assumption corresponds, for example, to the crystallization of rod-like particles where the rod cross section remains unchanged during growth and dissolution and crystals grow only in the length direction. It is however accurate for arbitrary geometries in the case the actual change of the second moment during an individual cycle is small. We verified the validity of the assumption through a broad set of numerical simulations that are presented in Supporting Information, Section S.2.

Introducing the following new variables and parameters (the condition that *m*_2,1_ > *m*_2,2_ introduces the asymmetry needed to trigger deracemization)
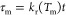
24
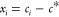
25
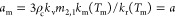
26
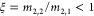
27
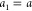
28
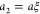
29yields the following pair of ODEs (we indicate the derivative of a variable with respect to the dimensionless time τ with a dot above the variable itself)
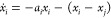
30

The system can conveniently be written in vector notation, with  and the matrix of coefficients, , defined below
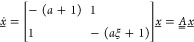
31

Note that .

Before studying the solution of this system, let us introduce the following parameter, which is bounded between 0 and 1
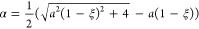
32

The two eigenvalues of the matrix  are
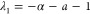
33
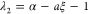
34with λ_1_ < λ_2_ < 0. The matrix  can be decomposed according to , whereby the eigenvectors of  are used as columns to define the orthogonal matrix 
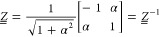
35

Note that  has been constructed such that it is symmetric as indicated by the second equality.  is a diagonal matrix, whose diagonal elements are λ_*i*_. Thus, the solution of the system of linear ODEs (eq 31) can be written for a generic initial condition  as
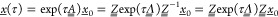
36where  is the matrix exponential that can be calculated using the matrix  and the diagonal matrix , whose diagonal elements are exp(τλ_*i*_).

#### 4.2.2 Temperature Cycles

Temperature cycles consist of alternate time periods spent by the system first at low temperature, *T*_g_, and then at high temperature, *T*_d_, i.e., under conditions first where the solution is supersaturated (*x*_*i*_ > 0) and crystals grow, and then where the solution is undersaturated (*x*_*i*_ < 0) and crystals dissolve, respectively. The transition from *T*_g_ to *T*_d_ and back occurs instantaneously; i.e., contrary to standard temperature cycles, there are neither heating nor cooling ramps. Since in practice, ramps are instrumental to minimize nucleation, the idealized model above, that does not include nucleation, is consistent with practice. The time periods spent at *T*_g_ and *T*_d_ are *t*_g_ = τ_g_/*k*_r_(*T*_g_) and *t*_d_ = τ_d_/*k*_r_(*T*_d_), respectively.

The general discussion above is specialized to the two operation modes through the following definitions.

During growth, at *T*_g_
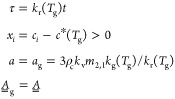
37

During dissolution, at *T*_d_
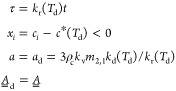
38

Iterating the solution given by eq 36 through the idealized temperature cycles, the system attains a cyclic steady state, where each cycle consists of alternate periods of growth and of dissolution (see Supporting Information, Section S.2.2 for a set of illustrative numerical simulations). Between the two modes, the values of *x*_*i*_ must be converted to account for the change of temperature hence of reference solubility; to this aim, we introduce the vector *s̲*, whose elements are the solubility difference Δ*c*_∞_ = c*(*T*_d_) – c*(*T*_g_). Using vector notation and starting from the initial state of growth, called , one obtains
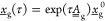
39
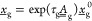
40
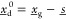
41
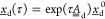
42
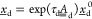
43
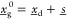
44where the last equation enforces the condition for the attainment of the cyclic steady state. Combining the equations above and solving for  yield an explicit equation for this state vector
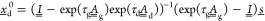
45where  is the unitary matrix. The vector  is the fixed point of the transformation defined by the sequence of eqs 39–44, whose existence is demonstrated by the fact that we can obtain an explicit expression for it, i.e., eq 45. From the last equation, one can also calculate the following explicit expression for the change in the state vector during dissolution
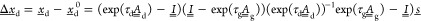
46

It is worth noting that the two components of this last vector are positive, as concentrations increase during dissolution, and that , as one can easily verify using the equations above.

#### 4.2.3 Exact Condition for Deracemization

We formalize the conditions for deracemization based on two considerations. First, in a process that reaches a cyclic steady state, such conditions must be valid for each individual cycle. Second, effective deracemization can be characterized in different ways, e.g., by looking at the evolution of the two populations of crystals (that of the target enantiomer must increase in number and size and vice versa) or by considering the solution: we follow the latter approach and recognize that the system deracemizes if and only if the net direction of the deracemization reaction during one entire cycle is from the minor to the major enantiomer. Such condition can be formalized as follows, where we use the following property of the matrix exponential, i.e., 
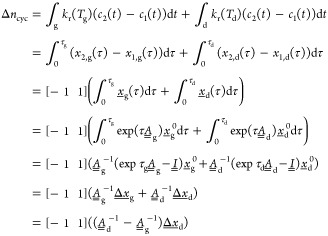
47

The last expression can be transformed and simplified, so that the condition for deracemization (Δ*n*_cyc_ > 0) reduces to the following inequality, written in vector notation first and then using scalar quantities
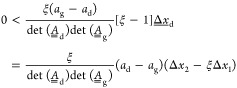
48

In the last expression, the fraction consists of positive quantities; the factor (*a*_d_ – *a*_g_) may be positive or negative; the last factor, i.e., (Δ*x*_2_ – ξΔ*x*_1_), appears to be ambiguous. However, this is the most remarkable result of this derivation, namely, that the last term is always positive, whatever the (positive) values of the six parameters that characterize the system, namely, *a*_g_, *a*_d_, ξ, τ_g_, τ_d_, and c*(*T*_d_) – c*(*T*_g_). As a consequence, the difference (*a*_d_ – *a*_g_) must also be positive to fulfill the conditions for deracemization, which can be simply written as
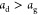
49

### 4.3 Experimental Section

#### 4.3.1 General Protocol

All experiments were conducted with the conglomerate-forming compound NMPA. NMPA was synthesized following the protocol outlined in our earlier work.^14^ NMPA racemizes in the presence of non-nucleophilic bases such as DBU.^4^ We used a mixture of 95/5 (w/w) isopropanol and acetonitrile (ACN) as the solvent, and *tert*-butyl methyl ether as the antisolvent to wash crystals after filtration, all in line with earlier work.^11,43^ Both DBU and the solvents were purchased from Sigma-Aldrich with a purity of 99% and used without further purification.

Saturated solutions of NMPA were prepared in an EasyMax 102 apparatus (Mettler Toledo) by adding an excess amount of NMPA to 100 g of the solvent mixture and stirring for 8 h to allow for equilibration at the target temperature of the experiment. For each deracemization experiment, 5 g (5.032 ± 0.0035 g) of saturated solution was transferred into 10 mL cylindrical glass vials (2 cm diameter and 10 cm height) using a syringe equipped with a hydrophilic syringe filter (PTFE, 0.22 μm, pk. 100) before DBU (6 μL g_s_^–1^) was added to each vial. In all experiments, the resulting mixture was stirred by using a magnetic stirring bar with 1000 rpm. An initial suspension density of 40.0 g kg_s_^–1^ was used, amounting to a mass of 0.2 g for each vial. 0.201 ± 0.0005 g of crystals was added to the crystallizers, and the temperature evolution was recorded through inserted K-type thermocouples. A single batch of NMPA crystals was prepared with an initial enantiomeric excess of 0.4 using the protocol explained in our earlier work^11^ and was used in all experiments reported here.

Samples were taken throughout each experiment by extracting 80 μL of suspension using a precision pipet. Crystals were collected from the suspension by vacuum filtration using a Büchner funnel and an MS PTFE membrane filter with a pore size of 0.45 μm. The crystals were then washed with few droplets of antisolvent to remove the potential residual of DBU. The dried samples were dissolved in ACN and analyzed with high-performance liquid chromatography according to the protocol reported earlier.^43^

#### 4.3.2 Temperature-Cycling Experiments

Three sets of temperature-cycling experiments were carried out with four vials each in the EasyMax 102 apparatus using the three temperature amplitudes of 2, 1, and 0.5 °C and an initial enantiomeric excess of ee_0_ = 0.4. The lower temperature of the cycle, i.e., the temperature of the growth step, was set to *T*_g_ = 40 °C in all experiments, hence the dissolution temperatures were *T*_d_ = 42 °C, *T*_d_ = 41 °C, and *T*_d_ = 40.5 °C, respectively. The cycles were designed such that dissolution and growth steps are of equal duration and that the total cycle time was 20 min (three cycles per hour); note that the actual cycle times as measured by the thermocouple turned out slightly longer for the experiments with 0.5 and 2 °C amplitudes, with values on the order of 21 min. Heating and cooling rates were set to 1.0 K min^–1^ for the experiments with 2 and 1 °C amplitude and to 0.3 K min^–1^ for the 0.5 °C experiment. We chose slower ramps for the 0.5 °C experiments to mitigate the issue of temperature overshooting. One vial per experiment was equipped with a thermocouple. Cycle efficiencies were computed for all temperature-cycling experiments, as discussed in the Supporting Information in Section S.1.

#### 4.3.3 Isothermal Experiments

Two sets of isothermal experiments were performed with four vials each. In the first, the temperature of the jacket surrounding the vials was controlled at 26 °C, and in the second, the temperature was not controlled and hence subject to the ambient conditions in the laboratory. For the controlled experiments, the vials were placed in the EasyMax 102 apparatus (jacket temperature set to 26 °C); in the second case, vials were placed on a magnetic stirring plate next to the window of the laboratory to allow for direct contact with sunlight. In the controlled experiments, one out of four vials was equipped with a thermocouple, and in the uncontrolled ones, all vials were equipped with a thermocouple each.

## Data Availability

All experimental data and code used in this work are available on request.

## Supporting Information Available

Methodology used to compute cycle efficiencies from experimental data and values obtained for experiments reported earlier^45^ and additional considerations related to the assumptions made in deriving the analytical solution; namely, the effect of a nonlinear driving force for crystal growth and dissolution, occurrence of the cyclic steady state, and effect of more complex cycle configurations (PDF)
