# Supplementary material for: Exact and Ubiquitous Condition for Solid-State Deracemization in Vitro and in Nature
Source: J Am Chem Soc. 2024 Feb 2;146(6):3872–82. doi: 10.1021/jacs.3c11332 (PMC10870780; doi:10.1021/jacs.3c11332)
Supplement: Supplementary file 1 — ja3c11332_si_001.pdf [file ja3c11332_si_001.pdf]

Supporting information for: Exact and Ubiquitous Condition for Solid-State Deracemization in Vitro and in Nature

Leif-Thore Deck, Mercedeh Sadat Hosseinalipour, and Marco Mazzotti*

Institute of Energy and Process Engineering, ETH Zurich, 8092 Zurich, Switzerland

E-mail: marco.mazzotti@ipe.mavt.ethz.ch

Abstract

This document contains Supporting Information (SI) for the manuscript *Exact and Ubiquitous Condition for Solid-State Deracemization in Vitro and in Nature*. Section S.1 explains the methodology used to compute cycle efficiencies from experimental data, and presents the values obtained for experiments reported earlier.[1] These values are discussed within the framework of the analytical solution derived in the main body of this work. Section S.2 reports additional considerations related to the assumptions made in deriving the analytical solution; namely the effect of a non-linear driving force for crystal growth and dissolution, the occurrence of the cyclic steady state, and the effect of more complex cycle configurations.

S.1 Experimental cycle efficiencies

S.1.1 Methodology

Here we explain how to obtain estimates of the cycle efficiency η for experimental temperature-cycling data. We first discuss the general approach, which we apply to a set of literature data, before estimating η for the experiments carried out in this work.

Breveglieri *et al.*[1] designed temperature profiles such that a certain fraction of the minority enantiomer dissolves in the first cycle. This is quantified by the dissolution factor δ_0:

$$\delta_0 = \frac{\rho_0(1-ee_0)}{2\Delta c_\infty} \tag{S1}$$

whereby the enantiomeric excess in the suspension is $ee = \frac{n_1-n_2}{n_1+n_2}$. Its value at the beginning of the experiment is $ee_0 = ee(t=0)$ and may be converted into an initial enantiomeric ratio ξ_0 (defined on a surface-basis) assuming that the initial particle size distributions of both enantiomers. ρ_0 denotes the total mass density of crystals in the suspension at the beginning, i.e., $\rho_0 = n_1(t=0)+n_2(t=0)$. The dissolution factor δ_0 is useful for this work, because it is linked to the total amount of material that must react towards enantiomer 1 to achieve homochirality, termed Δn_{tot}. All of enantiomer 2 except for the part that anyways dissolves during every cycle upon heating must be converted, so that $\Delta n_{\text{tot}} = n_2(t=0) - \Delta c_\infty$. We further assume that every cycle converts the same amount of material, so that $\Delta n_{\text{tot}} = \Delta n_{\text{cyc}} \times n_{\text{c}}$. This enables the definition of the experimental cycle efficiency η:

$$\eta = \frac{\delta_0 - 1}{n_{\text{c}}} \tag{S2}$$

This approach provides an easily accessible estimate of η from experimental data that just requires the values of n_{c} and δ_0. It is worth noting that this approach only considers the initial properties of the suspension; naturally, the value of ξ changes throughout the process as more and more material deracemizes. This is not considered here, nor is it taken account in the computation of η using the analytical solution (which provides η for a process that achieves cyclic steady-state at a constant

value of ξ). In fact, the decrease in ξ over time leads to an acceleration of the deracemization process, as shown in Figure 2 in the main body of this work. Given the large initial asymmetry used ($ee_0 = 0.4$) in the experiments, such effect is only of limited relevance and the estimated values of η can be considered as accurate determinants of the experimental deracemization performance.

S.1.2 Results

Here we compute the values of the cycle efficiency following the approach outlined in Section S.1.1. We first do so for for experiments involving the three model compounds N-(2-methylbenzylidene)-phenylglycine amide **(NMPA)**, 2- (benzylideneamino)-2-(2-chlorophenyl) acetamide **(CPG)** and 3,3-dimethyl-2-((naphthalen-2-ylmethylene)amino) butanenitrile **(tLEU)** reported previously.[1] All three compounds racemize in the presence of the non-nucleophilic base 1,8-Diazabicyclo[5.4.0]undec-7-en **(DBU)**. The overall suspension density, ρ_0, lies between 10 and 60 g/kg solvent, with ee_0 values of 0.4 in most experiments, corresponding to $\xi_0 = 0.43$. η was found to predominantly lie between 0.08 and 0.13 for all three model compounds.

Table S1: Analysis of experimental data reported by Breveglieri *et al.*[1]; the enumeration of the experiments equals the one of the referenced work. The model compounds studied are NMPA, CPG, and tLEU. The values of the initial enantiomeric excess, ee_0, of 0.4 and 0.6 correspond to values of the enantiomeric ratio ξ_0 of 0.43 and 0.25, respectively.

Exp.	Δc_∞ [g kg_s^{-1}]	ρ_0 [g kg_s^{-1}]	n_c [-]	δ_0 [-]	ee_0 [-]	η [-]
n1, NMPA	2.0	10	5	1.5	0.4	0.10
n2, NMPA	2.0	13	11	2	0.4	0.09
n3, NMPA	4.0	27	9	2	0.4	0.11
n4, NMPA	6.0	40	8	2	0.4	0.13
n5, NMPA	2.0	23	20	3.5	0.4	0.13
n6, NMPA	2.0	33	53	5	0.4	0.08
n7, NMPA	6.0	40	2	1.4	0.6	0.20
n8, NMPA	8.0	56	4	1.4	0.6	0.10
n9, NMPA	6.0	40	4	1.4	0.6	0.10
n10, NMPA	2.0	10	5	1.5	0.4	0.20
n11, NMPA	2.0	13	11	2	0.4	0.09
n12, NMPA	4.0	27	9	2	0.4	0.11
n13, NMPA	2.0	23	25	3.5	0.4	0.10
c1, CPG	2.0	10	16	1.5	0.4	0.03
c2, CPG	2.0	13	20	2	0.4	0.05
c3, CPG	4.0	27	10	2	0.4	0.10
c5, CPG	2.0	23	26	3.5	0.4	0.10
c11, CPG	2.0	13	15	2	0.4	0.07
c13, CPG	2.0	23	24	3.5	0.4	0.10
c14, CPG	4.0	47	18	3.5	0.4	0.14
t1, tLEU	2.0	10	5	1.5	0.4	0.10
t2, tLEU	2.0	13	9	2	0.4	0.11
t3, tLEU	4.0	27	7	2	0.4	0.14
t11, tLEU	2.0	13	10	2	0.4	0.10
t12, tLEU	4.0	27	12	2	0.4	0.08
t15, tLEU	4.0	20	5	1.5	0.4	0.10
t16, tLEU	4.0	20	7	1.5	0.4	0.07

Next, we estimate the cycle efficiency η for the temperature cycling experiments carried out in this work, which involve small temperature amplitudes of 2°C, 1°C, and 0.5°C. The corresponding dissolution factors were computed as outlined by Breveglieri *et al.*[1], and are 7.5, 15.2, and 30.7, i.e., much larger than those in the literature study.

For 2°C, all experiments exhibited complete deracemization after about 55 cycles; for 1°C, after 80 cycles; and for 0.5°C, after 160 cycles. This corresponds to cycle efficiencies of 0.12,

0.17, and 0.18, respectively. In all cases only little variability was observed among the four vials used in each experiment.

S.1.3 Discussion

To understand whether the experimental values of the cycle efficiency computed in Section S.1.2 are attainable through the newly identified mechanism, we evaluated the analytical solution of η for different values of the three parameters a_d, a_g, and ξ, as shown in Figure S1. This analysis focuses on the model compound NMPA, which has been studied intensely by our group,[1–3] and by others,[4,5] over the past decade. Motivated by the observation[1] that longer steps did not have a major effect on the cycle efficiency of NMPA temperature cycling experiments, we assume that the step durations t_d and t_g are sufficiently long that the concentration levels of the enantiomers in solution approach the equilibrium values, i.e., the solubility, before the steps end. In this case, η is independent of t_d and t_g. The lines shown in panel (a) correspond to different values of the ratio $a_\mathrm{d}/a_\mathrm{g}$ and to the experimental value of $\xi_0 = 0.43$. Even though the precise crystallization kinetics of the model compounds are not known, one can identify the space of parameter values where the analytical solution and experiments match each other in terms of η (colored regions, corresponding to the cycle efficiencies observed in the literature experiments). The red region that corresponds to small values of a_g can be ruled out, because a previous study has shown that an increase in the reaction rate (which would decrease the values of a_d and a_g) increases the deracemization rate and hence η.[3] Consequently, the relevant parameter space is given by the blue region.

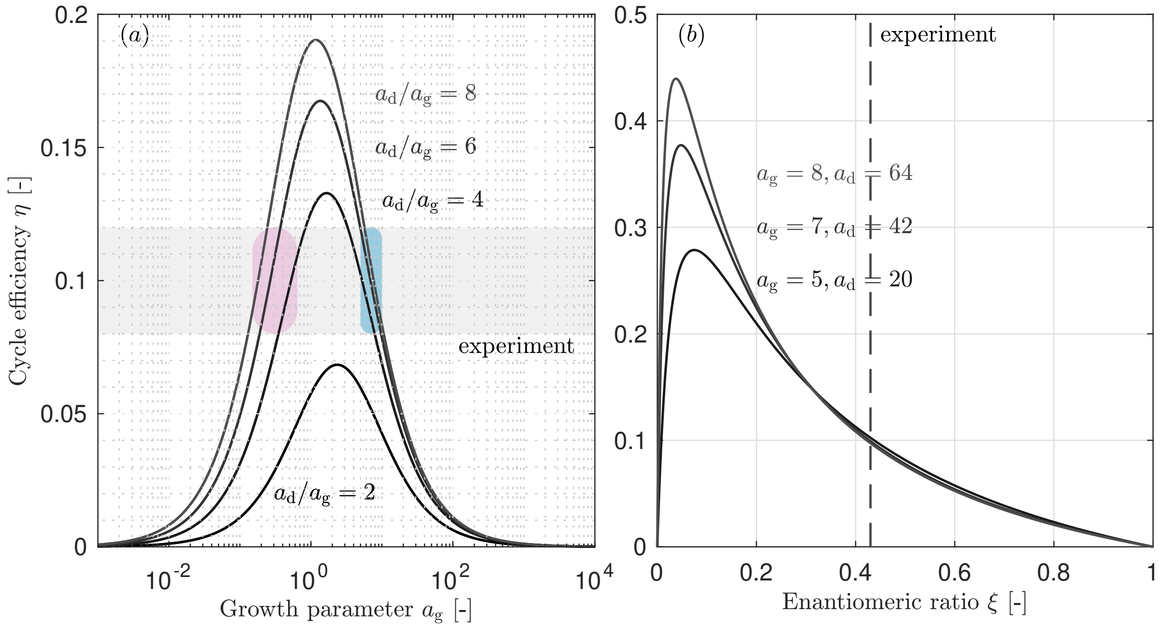

Figure S1: Values for the cycle efficiency given by the analytical solution in the limit of long step durations where η depends only on the three parameters a_g, a_d, and ξ. (a) η as a function of a_g with $\xi = 0.43$. The lines represent different values of the ratio a_d/a_g. (b) η as a function of ξ. The lines represent combinations of a_g, a_d that lead to model predictions that match the experimental value of η.

From a physical point of view, it is well plausible that the actual parameter values associated to the literature experiments lie within the identified space: first, the required values of the ratio $a_d/a_g \geq 4$ agree with the ratios of growth and dissolution rates reported in the literature for different compounds, including sodium chlorate ($k_d/k_g = 4$),[6] and paracetamol ($k_d/k_g > 2.5$)[7]. Second, given a characteristic time of the racemization reaction on the order of 10 min to an hour, and that crystallization is complete in step durations on the order of 10 min, it must hold that $a_g > 1$.

Considering the identified parameter space, one can explain the recent observation by Belletti *et al.*[5] that temperature-cycling experiments involving NMPA showed slower deracemization in some cases when they were combined with grinding. Grinding decreases the mean crystal size in the suspension, hence increasing the values of a_d and a_g, which in turn leads to smaller values of η under the relevant conditions. This experimental observation in fact cannot be explained when considering alternative deracemization mechanisms based on crystal ripening, since ripening becomes

more pronounced the smaller the crystal size. That grinding accelerates isothermal deracemization processes, on the other hand, may be due to the fact that the grinding process enhances the naturally occurring temperature fluctuations. Obviously, such enhancement is of little relevance in temperature cycling processes which are dominated by the periodic temperature variations.

Finally, S1 panel (b) illustrates the dependency of the cycle efficiency η on the enantiomeric ratio ξ for sets of realistic parameter values. η generally increases when decreasing ξ, except when ξ becomes very small values, i.e., under conditions where the suspension is already nearly homochiral. Keeping in mind that ξ decreases with every cycle during a successful deracemization process, it follows that deracemization is to accelerate throughout the process. This is the reason why the numerical simulations of solid-state deracemization reported in Figure 2 in the main body of this work exhibit an acceleration in the increase of the enantiomeric excess over time.

S.2 Additional considerations

In this section, we assess how the predicted deracemization performance changes when relaxing the assumptions made in deriving the analytical solution. We consider the role of the linear driving force for crystal growth and dissolution in Section S.2.1, the emergence of the cyclic steady state in Section S.2.2, and more complex cycle configurations such as those including temperature ramps in Section S.2.3.

S.2.1 Relaxing the linear driving force

In this section, we discuss the effect of a non-linear driving force for crystal growth and dissolution on deracemization. To this end, we numerically solved the general model as outlined in the Methodology section of the main body for values of the exponents $g = d = 1$ (termed 3D, $\ln(S)$). We further solved two simplified systems, one comprising 3D growth and the linearized driving force (termed 3D, Δc), and one comprising 1D growth and the linearized driving force (termed 1D, Δc); the latter corresponds to the assumptions made in the derivation of the analytical solution. In particular, 1D growth refers to the assumption that the surface area of the crystals does not change

as their volume changes, and 3D growth to the general case where the surface area does change. Figure S2 compares the predictions for the cycle efficiency η as a function of the parameter ratio $a_\mathrm{d}/a_\mathrm{g}$ obtained through the analytical solution and the three sets of numerical simulations. The values for η that belong to the simulations were obtained by simulating a single temperature cycle subject to sufficiently long step duration that the concentration levels of the enantiomers in solution approach the equilibrium values.

Panel (a) shows the result for cycling between the equilibrium concentrations of 20 g $\mathrm{kg_s^{-1}}$ and 25 g $\mathrm{kg_s^{-1}}$, whereas panel (b) shows the outcome for cycling between 20 g $\mathrm{kg_s^{-1}}$ and 20.2 g $\mathrm{kg_s^{-1}}$. All simulations were generated for a constant value of the growth parameter $a_\mathrm{g} = 5.15$ and for the surface area ratio $\xi_0 = 0.43$, while changing the value of the dissolution parameter.

All four solutions in both panels indicate that an increase in the ratio $a_\mathrm{d}/a_\mathrm{g}$ leads to a more positive value of the cycle efficiency. The exact solution (black dashed line) and the corresponding numerical solution (Sim. 1D, Δc, green) quantitatively agree with each other; this verifies the accuracy of the numerical implementation of the three numerical solutions. When comparing the exact solution with the numerical solution for 3D growth (Sim. 3D, Δc, violet), only minor differences are observed. Importantly, the root of η lies exactly at $a_\mathrm{d}/a_\mathrm{g} = 1$, hence the choice of 3D or 1D growth does not affect the condition for deracemization. The minor difference in the values of η results from the change in surface area of the crystals during growth and dissolution within a cycle that is considered in the 3D growth simulation, but not in the exact solution. Finally, the line corresponding to the solution with the non-linear driving force (Sim. 3D, ln(S), blue) exhibits a similar shape as the one for the linear driving force, but it is shifted towards the right, i.e., deracemization requires $a_\mathrm{d}/a_\mathrm{g} > 1$.

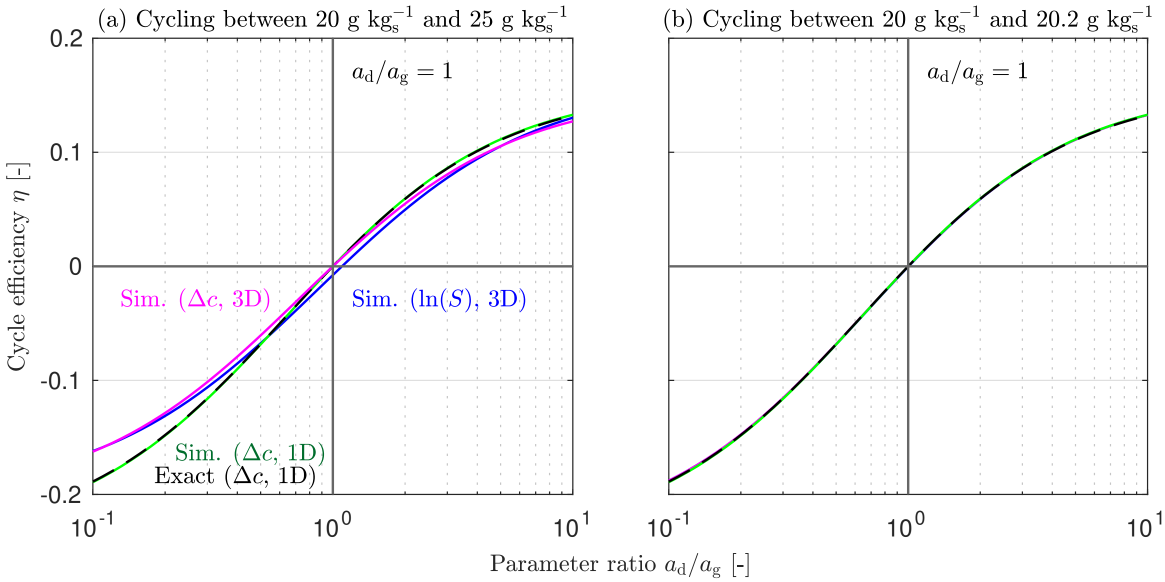

Figure S2: Comparison of the exact (black dashed lines) and three numerical solutions to temperature cycling-induced deracemization for two cases. The numerical solutions correspond (i) to the general case with logarithmic driving force and 3D growth (blue lines), (ii) to a simplified version considering 3D growth and the linear driving force (magenta line), and (iii) to the conditions of the analytical solution with 1D growth and linear driving force (green line). Panel (a): Cycling between solubilities of 20 g kg_s^{-1} and 25 g kg_s^{-1}. Panel (b): Cycling between solubilities of 20 g kg_s^{-1} and 20.2 g kg_s^{-1}. All simulations were generated for a constant value of the growth parameter $a_g = 5.15$ and for the initial enantiomeric ratio $\xi_0 = 0.43$, while changing the value of a_d.

This is because the logarithm introduces additional asymmetry to the system, since it is not symmetric around $S = 1$. Such asymmetry becomes more prevalent the larger the levels of super- and undersaturation at the beginning of the growth and dissolution steps. Panel (a), which shows an experimentally realistic case of temperature-cycling, highlights that such effect is small enough to be of limited practical relevance, i.e., the root of η lies at $a_d/a_g = 1.10$. In the case of a cycle with a small solubility difference, as illustrated in panel (b), all simulations perfectly overlap, as expected. Hence, the conclusions using the assumptions made in deriving the analytical solution can be generalized to systems with non-linear driving force and 3D growth.

S.2.2 The cyclic steady state

The analytical solution was derived under the assumption of a cyclic steady state, i.e., that the evolution of the concentration during a cycle is periodic, so that the concentration at the beginning of a cycle matches the one at the end. Such behavior is obvious for the case of sufficiently long growth and dissolution steps, where the concentration at the beginning of each step equals the solubility value of the step before. For short step durations, the situation is more complex and we show through numerical simulations (1D growth, Δc driving force) that a cyclic steady state emerges.

Figure S3 shows four sets of simulations: the top left panel shows simulations for sufficiently long cycles so that the concentration levels of the enantiomers (blue and red) approach the solubility (black) at the end of each cycle. This is similar to the simulations shown in Figure 1 in the main body of this work. The top right panel shows the evolution of the re-scaled concentration vector $\underline{x}$ with $x_i = c_i - c^*$: a value of [0 0] indicates that both concentration levels are at equilibrium. As initial condition, where the concentration levels are at 25 g $\mathrm{kg_s^{-1}}$ and the solubility at 20 g $\mathrm{kg_s^{-1}}$, the vector assumes values of [5 5], corresponding to the right top corner of the panel. During the growth step, the supersaturation depletes and the concentration levels approach 20 g $\mathrm{kg_s^{-1}}$, corresponding to a value of [0 0]. When the dissolution step starts, the solubility changes, so that $\underline{x}$ =[-5 -5]. During dissolution, the undersaturation is depleted and the concentrations again approach [0 0]; with the start of a new growth step, the solubility changes, leading to [5 5]. As can be seen clearly, the concentrations rapidly approach a cyclic steady state.

In the case that the dissolution step is short (row b), a similar behavior emerges, however, the value of $\underline{x}$ at the end of the dissolution step does not reach [0 0], but rather a value of [-1.8 -3.1]; the growth step hence starts at a value of [3.2 1.9]. Still, the growth step is sufficiently long that the equilibrium value of [0 0] is approached at its end. Just as in the first case, a cyclic steady state emerges here as well, and already between the second and third cycles no relevant difference in the concentration levels is observed. The same holds true in case the growth step is short, and dissolution is long (row c).

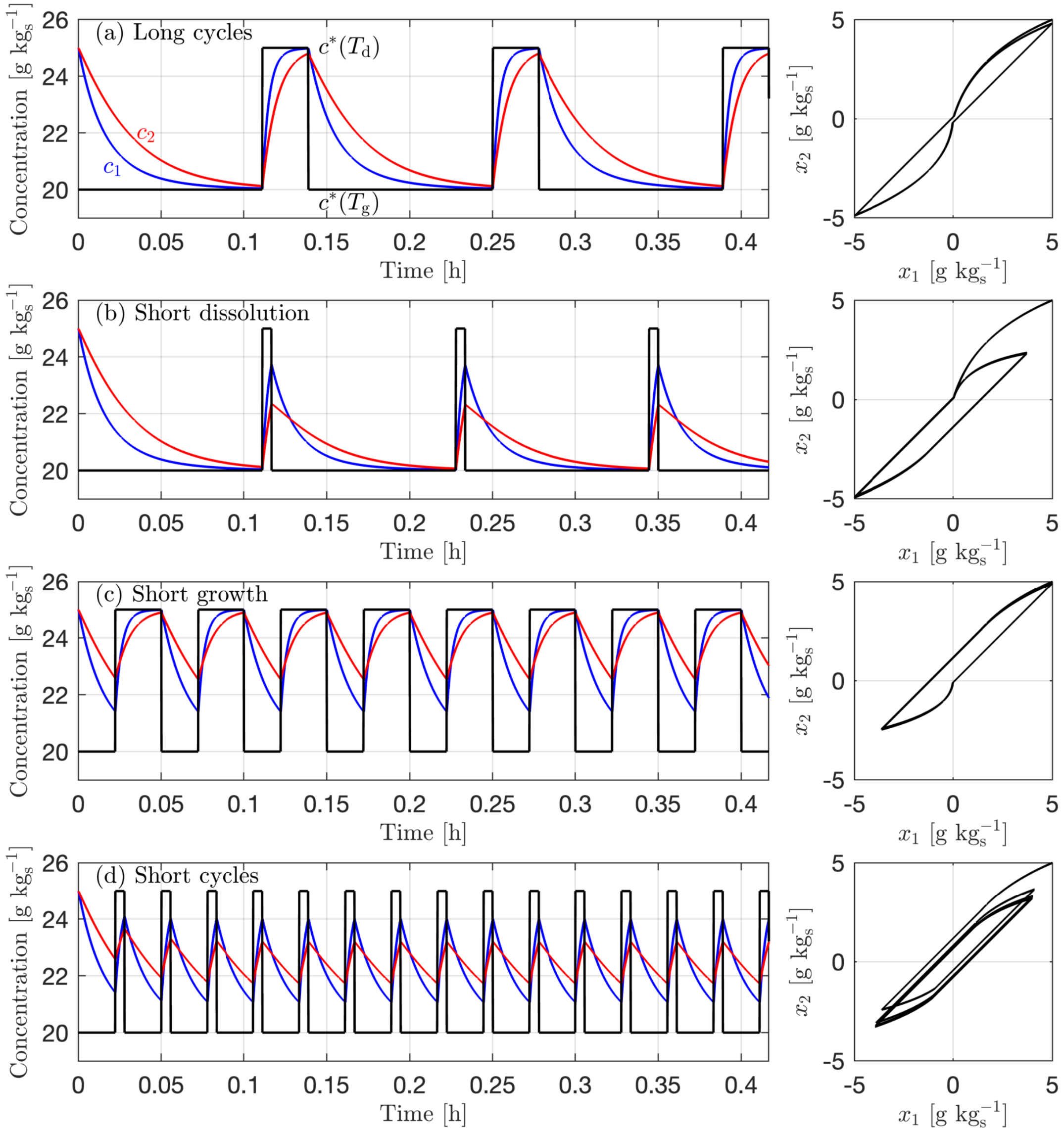

Figure S3: Numerical simulations for deracemization using four different step durations (a-d). The left panels illustrate the evolution of the concentration levels of both enantiomers in solution (red and blue), and the one of the solubility (black). The right panels show the cyclic evolution of the rescaled concentration vector $\underline{x}$ with $x_i = c_i - c^*$.

The final case that both steps are too short to approach the equilibrium is shown in the bottom row (d). Again, a cyclic steady state is reached, even though more slowly as in cases (b) and (c). These simulations clearly illustrate the emergence of the cyclic steady state independent of the

values of the step durations. Hence it is safe to contend that the assumption of such steady state in the derivation of the analytical solution is valid. Note that, while not shown explicitly, a similar behavior is observed when simulating the process using 3D growth and the logarithmic driving force.

S.2.3 Effect of more complex cycle configurations

We proved $a_\mathrm{d} > a_\mathrm{g}$ as exact condition for solid-state deracemization under the set of assumptions made to derive the analytical solution in the main body of this work, and we generalized this condition to systems with 3D growth and non-linear driving force for crystal growth and dissolution in Section S.2.1.

Here, we show that deracemization becomes feasible for $a_\mathrm{d} \leq a_\mathrm{g}$ when relaxing some further assumptions; namely when considering more complex cycle configurations combined with values of the exponents g and d in the expressions for the driving force that differ from 1. Figure S4 illustrates the deracemization performance for six simulations with $a_\mathrm{d} = a_\mathrm{g}$, i.e., for three cycle configurations with $g = d = 1$ (top row), and with $g = d = 2$ (second row). The columns report the cycle configurations; (a) is the base configuration with two steps connected through temperature jumps, (b) includes a third intermediate step for growth, and (c) shows a cycle comprising a dissolution step and a ramped growth step. To enable a fair comparison, the duration of a single cycle has been chosen to be the same in all three configurations, namely 12 min. While one may extend this study to an arbitrary number of cycle configurations, the three chosen ones sufficiently highlight the main effects.

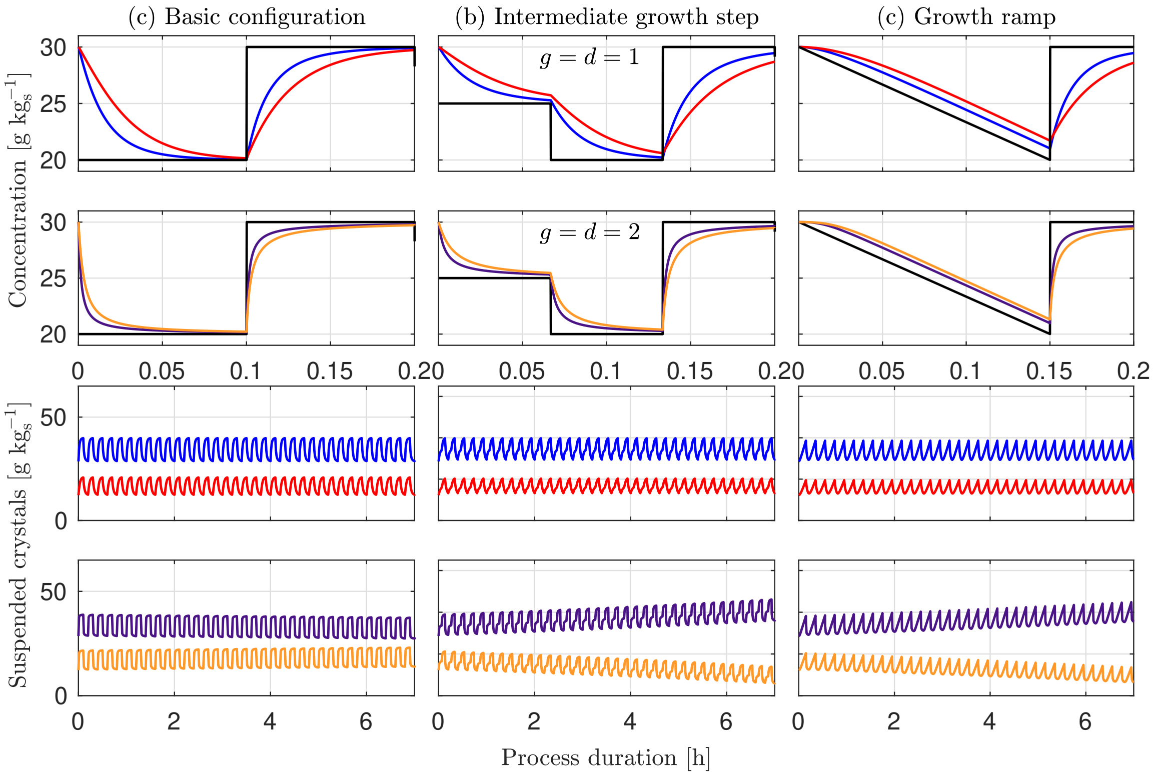

Figure S4: Numerical solution for temperature cycling-induced deracemization. Th red and blue lines show the evolution of concentration in the case of $g = d = 1$, the violet and yellow lines for $g = d = 2$. Three process configurations are considered (columns): (a) Basic cycle with one growth and one dissolution step. (b) Cycle with intermediate growth step. (c) Cycle with growth ramp and constant dissolution step. We note that for $g = d = 2$, configurations (b) and (c) enable deracemization; this is remarkable, since $a_\mathrm{d} = a_\mathrm{g}$, and hence the analytical solution would predict no change in the handedness of the suspension. The simulation parameters for the case $g = d = 2$ are reported in Table S2.

Following the exact condition for deracemization derived in the main body, the simulations should neither lead to deracemization nor to racemization. For $g = d = 1$, this is indeed the case for all three cycle configurations. For $g = d = 2$, however, the configurations with intermediate growth step and with growth ramp lead to deracemization. In these two cases, growth takes place at a lower value of the driving force than dissolution in average. For $g > 1$ this creates an additional asymmetry that shifts the process towards deracemization. This effect is most pronounced for a ramped step, where growth occurs at low, nearly constant supersaturation over the entire step dura-

tion. While not shown explicitly, similar effects are observed for configurations where dissolution occurs at lower values of the driving force than growth, which favors racemization.

It is worth underlining the practical relevance of this finding. The ratio a_d/a_g is hard to control in practice, as it depends on the physicochemical properties of the compound that is crystallizing, and on the temperature-dependency of the racemization reaction. In contrast, the manipulation of the cycle configuration provides an accessible means of improving the deracemization performance in the rather relevant scenario that at least one of the exponents g and d is unequal one.

Table S2: Set of parameter values used in the simulations for $g = d = 2$. Note that these simulations required a shorter time step due to the non-linearity introduced by the values of the exponents.

Parameter	Symbol	Values [unit]
Growth rate exponent	g	2 [-]
Dissolution rate exponent	d	2 [-]
Growth pre-factor at T_g (Δc driving force)	$k_g(T_g)$	$10^{-1.699}$[m s^{-1}kg^{-2}kg$_s^2$]
Growth/dissolution ratio at T_g	$k_d(T_g)/k_g(T_g)$	4 [-]
Time of growth step	t_g	variable
Time of dissolution step	t_d	variable
Simulation time step	t_{step}	0.05 [s]
Process duration	t_{tot}	until $N_2 = 0$

References

(1) Breveglieri, F.; Bodák, B.; Mazzotti, M. Deracemization via Periodic and Non-periodic Temperature Cycles: Rationalization and Experimental Validation of a Simplified Process Design Approach. *Organic Process Research & Development* **2021**, *25*, 2551–2565, DOI: 10.1021/acs.oprd.1c00310.

(2) Breveglieri, F.; Maggioni, G. M.; Mazzotti, M. Deracemization of NMPA via Temperature Cycles. *Crystal Growth & Design* **2018**, *18*, 1873–1881, DOI: 10.1021/acs.cgd.7b01746.

(3) Breveglieri, F.; Mazzotti, M. Role of racemization kinetics in the deracemization process via temperature cycles. *Crystal Growth & Design* **2019**, *19*, 3551–3558.

(4) Noorduin, W. L.; Izumi, T.; Millemaggi, A.; Leeman, M.; Meekes, H.; Enckevort, W. J. P. V.; Kellogg, R. M.; Kaptein, B.; Vlieg, E.; Blackmond, D. G. Emergence of a Single Solid Chiral State from a Nearly Racemic Amino Acid Derivative. *Journal of the American Chemical Society* **2008**, *130*, 1158–1159, DOI: 10.1021/ja7106349.

(5) Belletti, G.; Schuurman, J.; Stinesen, H.; Meekes, H.; Rutjes, F. P. J. T.; Vlieg, E. Combining Viedma Ripening and Temperature Cycling Deracemization. *Crystal Growth & Design* **2022**, *22*, 1874–1881, DOI: 10.1021/acs.cgd.1c01423.

(6) Fytopoulos, A. A.; Kavousanakis, M. E.; Gerven, T. V.; Boudouvis, A. G.; Stefanidis, G. D.; Xiouras, C. Crystal Growth, Dissolution, and Agglomeration Kinetics of Sodium Chlorate. *Industrial & Engineering Chemistry Research* **2021**, *60*, 7367–7384, DOI: 10.1021/acs.iecr.1c00595.

(7) Lovette, M. A.; Muratore, M.; Doherty, M. F. Crystal shape modification through cycles of dissolution and growth: Attainable regions and experimental validation. *AIChE Journal* **2011**, *58*, 1465–1474, DOI: 10.1002/aic.12707.
